# Recombinant Annexin A2 Administration Improves Neurological Outcomes After Traumatic Brain Injury in Mice

**DOI:** 10.3389/fphar.2021.708469

**Published:** 2021-07-12

**Authors:** Chongjie Cheng, Xiaoshu Wang, Yinghua Jiang, Yadan Li, Zhengbu Liao, Wenlu Li, Zhanyang Yu, Michael J. Whalen, Josephine Lok, Aaron S. Dumont, Ning Liu, Xiaoying Wang

**Affiliations:** ^1^ Department of Neurosurgery, The First Affiliated Hospital of Chongqing Medical University, Chongqing, China; ^2^ Neuroprotection Research Laboratory, Massachusetts General Hospital and Harvard Medical School, Charlestown, MA, United States; ^3^ Clinical Neuroscience Research Center, Department of Neurosurgery and Neurology, Tulane University School of Medicine, New Orleans, LA, United States; ^4^ Department of Pediatrics, Pediatric Critical Care Medicine, Massachusetts General Hospital and Harvard Medical School, Boston, MA, United States

**Keywords:** traumatic brain injury, Annexin A2, blood-brain barrier, cerebrovascular remodeling, angiogenesis, neurological outcomes

## Abstract

Microvascular failure is one of the key pathogenic factors in the dynamic pathological evolution after traumatic brain injury (TBI). Our laboratory and others previously reported that Annexin A2 functions in blood-brain barrier (BBB) development and cerebral angiogenesis, and recombinant human Annexin A2 (rA2) protected against hypoxia plus IL-1β-induced cerebral trans-endothelial permeability *in vitro*, and cerebral angiogenesis impairment of AXNA2 knock-out mice *in vivo*. We thereby hypothesized that ANXA2 might be a cerebrovascular therapy candidate that targets early BBB integrity disruption, and subacute/delayed cerebrovascular remodeling after TBI, ultimately improve neurological outcomes. In a controlled cortex impact (CCI) mice model, we found rA2 treatment (1 mg/kg) significantly reduced early BBB disruption at 24 h after TBI; and rA2 daily treatment for 7 days augmented TBI-induced mRNA levels of pro-angiogenic and endothelial-derived trophic factors in cerebral microvessels. In cultured human brain microvascular endothelial cells (HBMEC), through MAPKs array, we identified that rA2 significantly activated Akt, ERK, and CREB, and the activated CREB might be responsible for the rA2-induced VEGF and BDNF expression. Moreover, rA2 administration significantly increased cerebral angiogenesis examined at 14 days and vessel density at 28 days after TBI in mice. Consistently, our results validated that rA2 significantly induced angiogenesis *in vitro*, evidenced by tube formation and scratched migration assays in HBMEC. Lastly, we demonstrated that rA2 improved long-term sensorimotor and cognitive function, and reduced brain tissue loss at 28 days after TBI. Our findings suggest that rA2 might be a novel vascular targeting approach for treating TBI.

## Introduction

Traumatic brain injury (TBI) remains a significant source of death and permanent disability, contributing to nearly one-third of all injury-related deaths in the United States (Daniel Laskowitz, 2016). There are many pathophysiological mechanistic cascades in the dynamic brain damage and recovery processes after TBI. Emerging experimental investigations suggested microvascular failure is one of the key pathogenic factors, which essentially underlies the damaging secondary events that accompany traumatic brain injury (Lo, 2008; Adelson et al., 2011), featured in early capillary permeability disruption results in producing cerebral edema, inflammation, and secondary neuron death (Shlosberg et al., 2010; Simard et al., 2010). At delayed phases, vascular remodeling, particularly in angiogenesis and enhanced endothelial-derived trophic factor secretion, may promote endogenous neurogenesis and neuronal repair (Schwarzmaier et al., 2010; Xiong et al., 2010). Hence, cerebrovascular therapy targeting early BBB damage-mediated tissue injury, as well as subacute/delayed cerebrovascular remodeling-associated regeneration and repair, might have the advantage of being a wider treatment window and optimal effect over a prolonged period after TBI(Diaz-Arrastia et al., 2014).

Annexin A2 (ANXA2) is a 36KD cell membrane protein that has been proposed to be a key regulator of many biological processes (Dallacasagrande and Hajjar, 2020). One important function of ANXA2 is the modulation of BBB permeability. ANXA2 has been identified as an endothelial membrane F-actin binding protein in bridging junction formation (Lee et al., 2004), and plays central roles in modulating tight junction integrity and BBB permeability (Hayes et al., 2004; Hayes et al., 2009; Grieve et al., 2012). In our recent study, we showed for the first time that ANXA2 is an essential membrane protein that contributes to BBB development and integrity, as ANXA2 reduction leads to decreased expression of tight junction proteins ZO-1, Claudin-5, and adherens junction protein VE-cadherin in isolated cerebral microvessels fragments and the human brain microvascular endothelial cells (HBMEC), suggesting that the unique role of ANXA2 in BBB formation and function. Moreover, we further demonstrated that ANXA2 maintains trans-endothelial tightness *via* modulating the Robo4-paxillin-ARF6 (ADP-ribosylation factor 6) signaling pathway and deactivate ARF6 (Li et al., 2019). In addition, ANXA2 also plays a crucial role in promoting angiogenesis under physiological and pathological conditions (Liu and Hajjar, 2016). It has been reported that knockdown of ANXA2 inhibits cell division and proliferation (Chiang et al., 1999). Importantly, ANXA2 knock-out mice display postnatal angiogenesis impairments (Huang et al., 2011), which can be corrected by infusion of rA2 (Jacovina et al., 2009).

Based on these emerging experimental investigations, we hypothesized that ANXA2 might be one of the novel candidates for the development of cerebrovascular therapy that targeting post-TBI early BBB integrity disruption, as well subacute/delayed cerebrovascular remodeling, ultimately improve neurological outcomes after TBI. In the present proof of concept pilot study, we tested our hypothesis in a traumatic brain injury model (with the controlled cortex impact device) of mice. The effects of rA2 administration in vascular targeting effects of early BBB damage and late cerebrovascular angiogenesis and neurological outcomes were examined.

## Materials and Methods

### Production of rA2

rA2 was expressed in an *Escherichia coli* batch fermentation process, starting from a research cell bank using LB-glucose medium with IPTG induction as we previously described (Zhu et al., 2010). Briefly, the soluble protein was purified using a combination of hydrophobic interaction chromatography, ion-exchange chromatography, and hydroxyapatite chromatography, produced in the Bioexpression and Fermentation Facility at the University of Georgia (http://bff.uga.edu/). The final rA2 purity is 96% (endotoxin 0.5 EU/mg) with a concentration of 8 mg/ml.

### Traumatic Brain Injury in Mice

All procedures followed institutional and United States National Institutes of Health (NIH) guidelines and were approved by the Institutional Animal Care and Use Committee (IACUC) at Massachusetts General Hospital and Tulane University School of Medicine. The standard controlled cortical impact (CCI) model of traumatic brain injury (TBI) was performed in 12-week-old male C57 BL/6 J mice (Jackson Laboratory) as we previously described (Choi et al., 2016; Chung et al., 2019). Briefly, the mice were anesthetized with 2% isoflurane, and the mice head was secured in a stereotactic frame. During surgery, Anesthesia was maintained under 1.5% isoflurane with a face mask in 70% N_2_O and 30% O_2_ using a Fluotec 3 vaporizer (Colonial Medical Amherst, NH). After a midline longitudinal incision, a left parietal craniotomy (5.0 mm diameter) was performed between bregma and lambda with a portable trephine drill. Subsequently, the controlled cortical impact (CCI) was induced using a 3 mm diameter convex tip, at 5.0 m/s with 40 ms dwell time and 0.6 mm depth, mimicking a moderate TBI (Bajwa et al., 2016). The scalp was sutured closed, and the mice were then returned to their cages to recover from anesthesia. Sham-treated mice underwent craniotomy but not impact. All TBI mice were ear-tagged and assigned randomly into equal-sized groups, blinded to the investigators we previously described (Liu et al., 2019).

### Experimental Design and rA2 Administration

In the present study, we designed three sets of experiments to test our hypothesis. In the first set of experiments, we performed dose-range and time window effects of rA2 administration in early BBB integrity disruption by Evans blue extravasation assay. A previous study had reported that AXNA2 is administered into the spinal cord injury rat model *via* intrathecal injection (i.t.) with a dose of 100 μg/rat (250–300 g) (Fang et al., 2011). Considering the effectiveness of i.t may be lower than intraperitoneal injection (i.p.), the doses of rA2 ranged from 0.75 to 1.5 mg/kg were used to determine BBB leakage with Evans blue assay. We then tested the effects of optimal dose of rA2 (i.p. 1 mg/kg) treated at 2 h after TBI in the regulation of cerebrovascular endothelium adherens and tight junction expression by Western blot analysis at 24 h after TBI. In the second set of experiments, animals were treated with 1 mg/kg of rA2 (i.p.) initiated at 2 h after TBI and repeated daily for 7 days. Bovine Serum Albumin (BSA, 1 mg/kg) was used as vehicle control treatment at 2 h after TBI (TBI+vehicle).

### Enzyme-Linked Immunosorbent Assay

Blood samples were obtained from the eye veins of the anesthetized mice at 0, 2, 6, 12, 24, 48, and 72 h after i.p. administrated with 1 mg/kg of rA2, and centrifuged at 2,000 rpm for 15 min at 4°C. The concentration of ANXA2 in the collected plasma was then measured using the Human Total Annexin A2 DuoSet IC ELISA kit (Novus Biologicals, DYC3928-2) and calculated based on standard curves with purified rA2. The absorbance was measured at 450 nm with a microplate reader (Molecular Devices). The plasma ANXA2 half-life was calculated using the following the formula as described previously (Nilsen et al., 2020): t_1/2_ = log 0.5/(log Ae/A0)× t, where t_1/2_ is the half-life, Ae is the amount of ANXA2 remaining, A0 is the amount of ANXA2 on 2 h, and t is the elapsed time.

### Evans Blue Extravasation Assay for Blood-Brain Barrier Leakage Assessment

The integrity of BBB was investigated by measuring the extravasation of Evans blue at 24 h after injury following our previously published protocol (Aoki et al., 2002). Briefly, at 23 h post-TBI, mice were given with Evans blue dye (2% wt/vol, 200 μL in Saline) by tail vein injection. After circulation for 1 h, mice were deeply anesthetized and transcardially perfused with 0.1 mol/L ice-cold phosphate-buffered salines (PBS). The mice were decapitated, and brains were removed, weighed, and homogenized in 1.0 ml of trichloroacetic acid (50% in pure water), and centrifuged at 10,000 rpm for 20 min. The fluorescence in supernatants was read by a microplate reader (SpectraMax M5, Molecular devices) at 630 nm for excitation and 680 nm for emission. The Evans blue concentrations were normalized based on the results for the sham-operated brain hemispheres. The amount of Evans blue was quantified according to Evans blue external standard curve (25–2,000 ng/ml) in 50% TCA/ethanol (1:3), and expressed as nanograms of Evans blue per gram of brain tissue.

### Western Blot Analysis

Western blot analysis was followed by the standard protocol as we previously described (Choi et al., 2016). Briefly, following transcardial perfusion with 0.01 M PBS at pH 7.4, peri-lesion cerebral cortex and sub-cerebral cortex tissues were collected for protein extraction. The cortex tissues or cells were homogenized in lysis buffer (Cell signaling Technology) with protease inhibitors (Thermo Fisher Scientific), and the proteins were quantified with the BCA method with Pierce ™ BCA protein assay kit (ThermoFisher Scientific, 23,225). Denatured protein samples were separated by 10% SDS-PAGE and transferred to a polyvinylidene difluoride (PVDF) membrane. Membranes were blocked with 5% nonfat milk for 1 h and incubated overnight at 4°C with the following primary antibodies: AnxA2 antibody (1:500, Santa Cruz, sc-28385), ZO-1 rabbit polyclonal antibody (1:1,000, Invitrogen, #61-7300), Occludin mouse monoclonal antibody (1:1,000,Invitrogen, #33-1500), Claudin 5 mouse monoclonal antibody (1:1,000,Invitrogen, #33-2500), VE-cadherin rabbit polyclonal antibody (1:1,000, Invitrogen, #36-1900), VEGF antibody (1:500, Santa Cruz, sc-152), BDNF rabbit antibody (1:1000, Abcam, ab108319), Phospho-CREB (Ser133) (87G3) rabbit antibody (1:1000, Cell signaling, 9198S), and β-Actin mouse monoclonal antibody (1:3,000, Sigma, #A2228) overnight at 4°C. After extensive washing with 1 × TBST [(Tris-buffered Saline plus Tween) (1:1000)], the membranes were incubated with IRDye 800CW goat anti-Rabbit (1:10,000, LI-COR Biosciences, #926-32211) or Goat anti-Mouse IgG StarBright Blue 700 (1:10,000, Bio-Rad, #12004159) antibodies for 1 h. Images were captured with Bio-Rad ChemiDocTM MP Imaging System. Quantitative densitometry was performed on the protein bands by using Image J software. β-actin was served as equal loading controls.

### Isolation of Brain Microvessels Fragments

Brain microvessels were isolated as we described previously (Guo et al., 2016; Liu et al., 2019). Briefly, mice were deeply anesthetized and sacrificed at 24 h after TBI, and perfused transcardially with ice-cold PBS at pH 7.4. The mice were decapitated, and brains were removed. Ipsilateral and contralateral hemispheres were separated. Next, the cerebellum and the underlying white matter were removed, and the cortex was further rolled on the filter paper to remove big vessels. Collected cortical tissues from each hemisphere were homogenized on ice with Knote Dounce glass tissue grinder (Part 885300-0002; Kimble Chase Life Science, Vineland, NJ, United States) in ice-cold PBS and further centrifuged at 4°C, 1000 × g for 5 min. The pellet was further re-suspended with 18% Dextran solution (molecular weight 60–90 kDa; USB Corporation, Cleveland, OH, United States) in PBS and then centrifuged at 4°C, 2000 × g for 15 min to carefully separate the myelin and subsequently collect the vessel pellet. The vessel pellet was washed with 18% Dextran solution and further washed with PBS and filtered through a 40 μm cell strainer to get rid of the debris. The micro-vessels on the top of the cell strainer were used directly for RNA extraction.

### Reverse Transcription and Real-Time Polymerase Chain Reaction Assay

Reverse transcription and real-time polymerase chain reaction assay were followed by the standard protocol as we previously described (Liu et al., 2019). Total RNA was extracted with miRNeasy micro kit (Qiagen, Germantown, MD, United States) according to the manufacturer’s instructions and reverse-transcribed with QuantiTect Reverse Transcription Kit (Qiagen) as previously described (Andre et al., 2014). Real-time polymerase chain reaction (RT-PCR) was performed on an ABI 7500 Fast Real-Time PCR system using Taqman gene expression assays for multiple target genes listed as follows: Angpt1 (Mm00456503_m1), Tie2 (Mm00443243_m1), Vegf (Mm00437306_m1), Igf1 (Mm00439560_m1), Bdnf (Mm01334043_m1), Hprt (Mm01545399_m1). Reactions were performed in duplicate according to manufacturer instructions. Relative expression levels were measured with the 2^−ΔΔCt^ method and presented by fold change.

### MAPKs Proteome Profiler Array

The MAPKs proteome profiles were analyzed using the Proteome Profiler Human Phospho-MAPK Array Kit (R&D Systems, ARY002B) according to the manufacturer’s specifications. Briefly, 200 μg protein of HBMEC treated with PBS or rA2 were incubated for 1 h at room temperature with the supplied reconstituted Detection Antibody Cocktail. The array membranes were blocked with the blocking buffer, followed by incubating with the lysate-antibody mixtures overnight at 4°C on the platform shaker. After washing in 1× wash buffer, the array membranes were further incubated with Streptavidin-HRP in array buffer for 30 min at room temperature before mixing with the Chemi reagent mix. Images were then captured with Bio-Rad ChemiDoc™ MP Imaging System. Image J was used to quantify and determine spot density.

### WST Assay

The viability of cultured HBMEC was determined by WST assay in 48-well plates as we previously described (Zhao et al., 2019) with slight modification. Briefly, WST-1 assay solution (20 μL) was added to each well (containing 180 μL medium) and incubated for 3 h at 37 °C. Absorbance was determined at 490 nm against untreated cells using a microplate reader (SpectraMax M5, Molecular Devices) according to the manufacturer’s instruction.

### Cerebral Angiogenesis and Vessel Density Assessments

For cerebral angiogenesis measurement, mice were initially given 50 mg/kg of 5-bromo-2-deoxyuridine (BrdU; Abcam, Cambridge, MA) *via* intravenous administration at 7 days after TBI, followed by twice-daily treatment for seven consecutive days with 8–12 h interval prior to the sacrifice. Subsequently, mice were deeply anesthetized with isoflurane and perfused transcardially with PBS, followed by 4% paraformaldehyde perfusion. The brain was removed and fixed in 4% paraformaldehyde at 4°C for 24 h. The brains were then transferred to 30% sucrose solution at 4°C until it sank to the bottom of the tube. After dehydration, 20 μm sections of the hippocampus were collected using a freezing microtome (Leica) and stored at −80°C. Immunostaining was conducted using the standard protocol as we described previously (Choi et al., 2016). Brain sections were incubated in PBS containing 0.1% Triton X-100 for 1 h followed by incubation in 5% fetal bovine serum for 1 h. After that, the sections were incubated overnight in PBS containing antibodies mouse anti-BrdU (1:200, Santa Cruz, Dallas, TX), goat anti-CD31 antibody (1:200, R&D, Minneapolis, MN) at 4°C. On the following day, sections were washed with PBS, then incubated with secondary antibody conjugated to fluorescein (1:200, Invitrogen) for 1 h at room temperature. Sections were washed with PBS and then mounted with vectashield mounting medium containing DAPI after drying. For vessel density assessment, lectin dye (0.2 mg/ml) in a volume of 300 μL was given by tail vein injection and allowed to circulate for 10 min before sacrifice at 28 days after TBI. Fluorescence images were obtained using a fluorescence microscope (Nikon, Tokyo, Japan). Image fields include peri-lesion cortex, subventricular zone (SVZ), and dentate gyrus (DG) in the hippocampus in the ipsilateral and contralateral hemispheres of three consecutive brain sections were analyzed for each animal separately by an investigator blinded to experimental groups. Cell counts and quantification of fluorescence intensity/area were performed by NIH Image J software (Bethesda, MD).

### Primary Human Brain Microvascular Endothelial Cell Cultures and *In vitro* Angiogenesis Assays

Primary human brain microvascular endothelial cells (HBMEC) were purchased at passage 3 from Cell System (ACBRI 376 V) and used at passages 7–10 as we previously described (Wang et al., 2003). HBMEC were grown in endothelial complete medium (EndoGRO-MV-VEGF Complete Culture Media Kit, EMD Millipore, SCME003) to about 100% confluence and then insulted with hypoxia (with a modular chamber (Billups-Rothenberg)) perfused with 90% N_2_/5% CO_2_/5% H_2_ for 30 min at 37°C) for 24 h. For rA2 or rA2 plus KG-501 (2-naphthol-AS-E-phosphate, Selleckchem, S8409) co-treatment, HBMEC were cultured in endothelial medium containing 1% FBS to 80% confluence and then treated with rA2 plus DMSO or KG501 for 24 h.

Tube formation and scratch migration assays were used as indirect *in vitro* assays to assess angiogenesis as we previously described (Xing et al., 2009). For tube formation assay, HBMEC were harvested with trypsin, and 4 × 10^4^ cells were re-suspended in 300 µL of endothelial cell growth medium and treated with PBS, drA2, or rA2 (2 µg/ml) before plating onto 24-well plates coated with growth factor reduced Matrigel (Corning), and incubated at 37°C incubators for 24 h. The number of tubes was counted in three random fields from each well using an inverted phase-contrast microscope. For the scratch migration assay, HBMEC were plated on six wells plates at 90% confluence. The cells were scratched using a sterile 200 μL pipette tip, and the debris was removed by rinsing with the warm medium. The cells were treated with PBS, drA2, or rA2 (2 µg/ml) at 37°C for 24 h, then rinsed with PBS, followed by fixing with 4% PFA. The distance of the scratch closure was examined at 0 and 24 h by inverted phase-contrast microscopy at a magnification of 200×. Two areas were randomly selected and imaged in each well. Data were analyzed as a percentage of the initial gap area.

### Neurobehavioral Tests

For neurobehavioral texts, mice were initially given 1 mg/kg of rA2 *via* intraperitoneal injection at 2 h after TBI, followed by once-daily treatment for 7 days. Before and on days 1, 3, 7, 14, and 28 after TBI, sensorimotor function deficits were examined with modified NSS score, and Rota-rod tests followed the methods as we previously described (Choi et al., 2016; Liu et al., 2019). Briefly, for modified NSS, mice were used to perform 10 different tasks, and one score point was awarded for the inability to perform each of the tasks. For the Rota-Rod test, at the day before surgery, mice were trained on the Rota-rod (Harvard Apparatus, Holliston, MA, United States) for three consecutive trials at a slow rotational speed (4 rpm/min) for 1 min, followed by four trials with an accelerating rotational speed (from 4 to 40 rpm in 5 min) to obtain the baseline. On each testing day, the mice were tested with four accelerating trials with an inter-trial interval of 20 min. The average latency to the first fall of the rod was recorded.

Spatial learning and memory were assessed using a Y-maze test at 21 days after TBI as we previously described (Liu et al., 2019; Sirbulescu et al., 2019). Briefly, the Y-maze apparatus (395 mm in length, 120 mm in height, at 120°angles to each arm) was made of solid gray plastic and consisted of three arms. The mice were placed in the center of the apparatus and allowed to explore it for 5 min, which was captured by a digital video. The number of arm entries and the number of triads (a set of the successive entry of the mouse into three arms, e.g., triplets of ABC, BCA, CAB, etc.) were counted. The alternation behavior percentage (%) was calculated by the equation: % alternation = [(number of alternations)/(total arm entries minus 2)] × 100. A higher % alternation was considered indicative of improved cognitive performance.

### Quantification of Traumatic Brain Tissue Loss

At 28 days after neurobehavioral tests, mice were deeply anesthetized and sacrificed for quantitation of traumatic brain tissue loss by following a standard method as we previously described (Liu et al., 2019). Briefly, mice were anesthetized and transcardially perfused with chilled (4°C) PBS, pH7.4, followed by 4% paraformaldehyde in 0.1M PBS. Frozen brains were sectioned with a microtome, and six equally-spaced slices (50-μm-thick) from bregma: +1.54 to −3.46, with 1000 μm interval. Coronal brain sections were stained with Alexa Fluor® 488 conjugated microtubule-associated protein 2 (MAP2) antibody (1:200, MAB3418X, Sigma-Millipore, Burlington, MA, United States), and the images of brain sections were captured with an image scanner. The brain tissue loss was quantified with a standard computer-assisted image analysis program (Image J), and calculated with the following formula: Brain tissue loss = contralateral hemisphere volume- ipsilateral hemisphere volume.

### Statistical Analysis

All data are expressed as mean ± SEM. For parametric and continuous variable measurements such as BBB permeability, protein expression levels, tissue loss analysis, immunostaining assays, tube formation, and scratch migration assays, statistical analyses between two groups were determined by student’s t-test, and multiple groups were determined by one-way or two-way analysis of variance (ANOVA), followed by the Tukey *post hoc* multiple comparisons test. For NSS and Rota-rod, two-way (treatment × time) repeated-measures ANOVA analysis, followed by the Tukey *post hoc* multiple comparisons test. A *p*-value of less than 0.05 was considered statistically significant.

## Results

### ANXA2 Levels in Mice After i.p. Administration of rA2

C57BL/6 mice were subjected to i.p injection of rA2 (1 mg/kg). At 0, 2, 6, 12, 24, 48 and 72 h after injection, ANXA2 levels in plasma were measured using ELISA. The results showed that the peak of ANXA2 concentration (52 ± 14.58 ng/mg plasma) was observed at 2 h post-injection. The half-life is 8.9 h (Supplementary Figure 1).

### rA2 Treatment Reduces BBB Leakage After Traumatic Brain Injury in Mice

**FIGURE 1:**
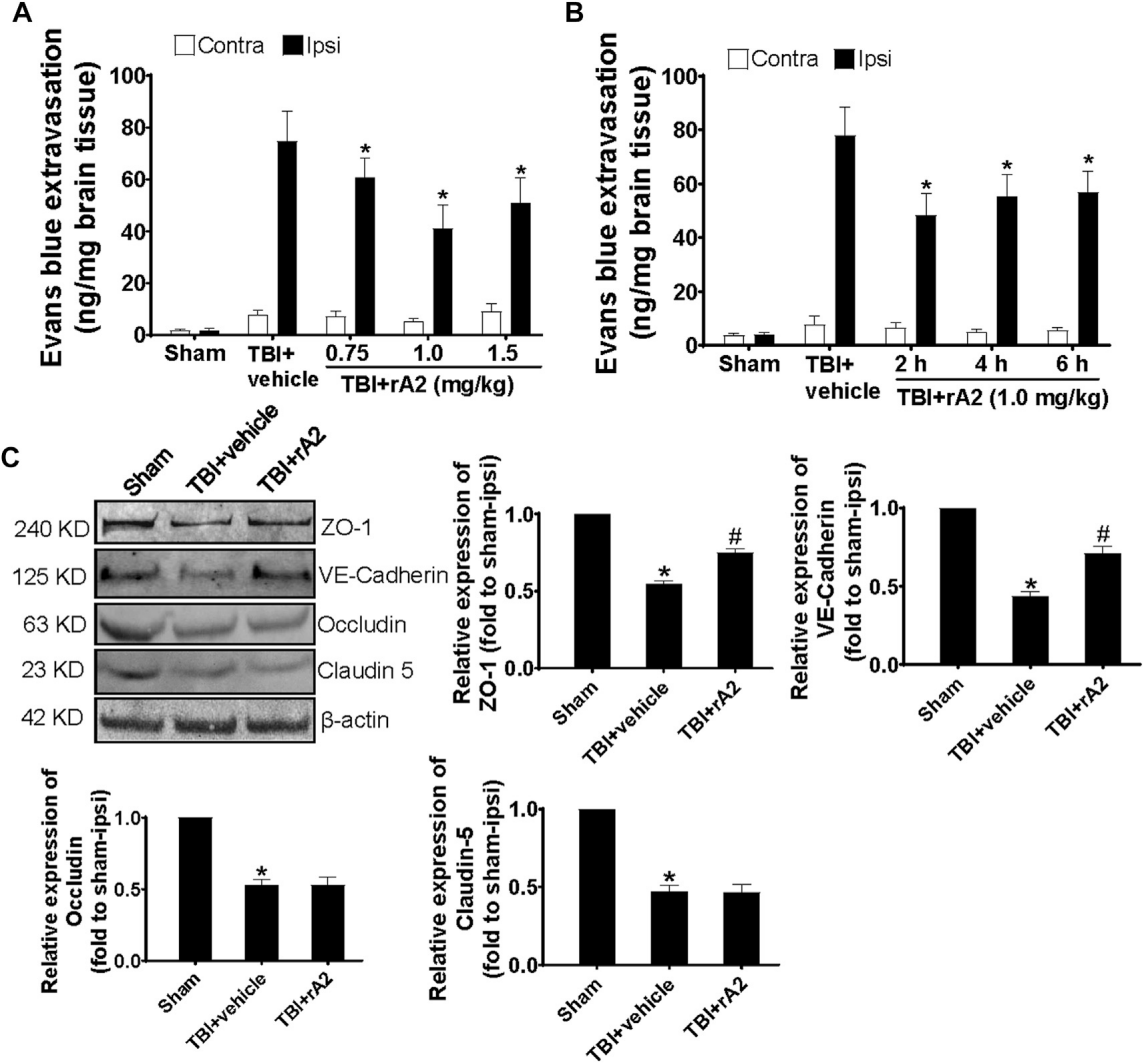
rA2 administration reduces BBB leakage after TBI in mice. **(A)** Dose-range (0.75, 1.0, and 1.5 mg/kg) effects of rA2 administration treated at 2 h in BBB permeability examined at 24 h after TBI. **(B)** The therapeutic time window of rA2 administration in BBB permeability reduction examined at 24 h after TBI, treated at 2 h, or 4 h, or 6 h after TBI, respectively. Data are expressed as mean ± SEM, *n* = 8 mice per group, *p < 0.05 vs. BSA treated ipsilateral hemisphere. **(C)** Representative gel images of western blotting and quantitative analysis for ZO-1, VE-Cadherin, Occludin, Claudin-5 expression in the ipsilateral hemispheres collected at 24 h after TBI. β-actin was used as the equal loading control. Data are expressed as mean ± SEM, *n* = 4 mice per group, *p < 0.05 vs. Sham, ^#^ *p* < 0.05 vs. TBI+vehicle (1 mg/kg BSA).

To determine the beneficial effects of rA2, we firstly asked whether rA2 protein may preserve protective effects against TBI-induced BBB damage of adult male mice. We tested the dose-range effect of rA2, and found at 24 h after TBI, Evans blue leakage was significantly decreased by 18, 35, and 25% in adult male mice treated (intraperitoneal injections) with 0.75, 1.0, and 1.5 mg/kg of rA2 at 2 h after TBI, respectively (Figure 1A), suggesting rA2 at 1 mg/kg was the optimal dose. We next tested the time window effect of rA2 at 1 mg/kg, and experimental results showed rA2 significantly decreased BBB leakage by 33, 23, and 19%, when treated at 2, 4, and 6 h after TBI, respectively, compared to TBI (TBI+vehicle) controls (Figure 1B). To examine the effect of rA2 administration in the regulation of adherens and tight junction expression, western blot analysis was performed to quantify the protein expression level of tight junction proteins ZO-1, occludin, claudin-5, and adherens junction protein VE-cadherin in the brain tissues at 24 h after TBI. Our results showed rA2 treatment (1 mg/kg) significantly ameliorated the TBI-induced down-regulation of ZO-1 and VE-cadherin (Figure 1C). These results suggest rA2 has a potent BBB protection effect, and a long therapeutic time window up to 6 h after TBI in mice, in part by prevention of TBI-induced down-regulation of cerebrovascular endothelium adherens and tight junction expression.

### rA2 Administration Augments Expression of Pro-Angiogenic and Trophic Factors in the Isolated Cerebral Microvascular Fragments and Brain Tissues at 7 Days After Traumatic Brain Injury in Mice

**FIGURE 2:**
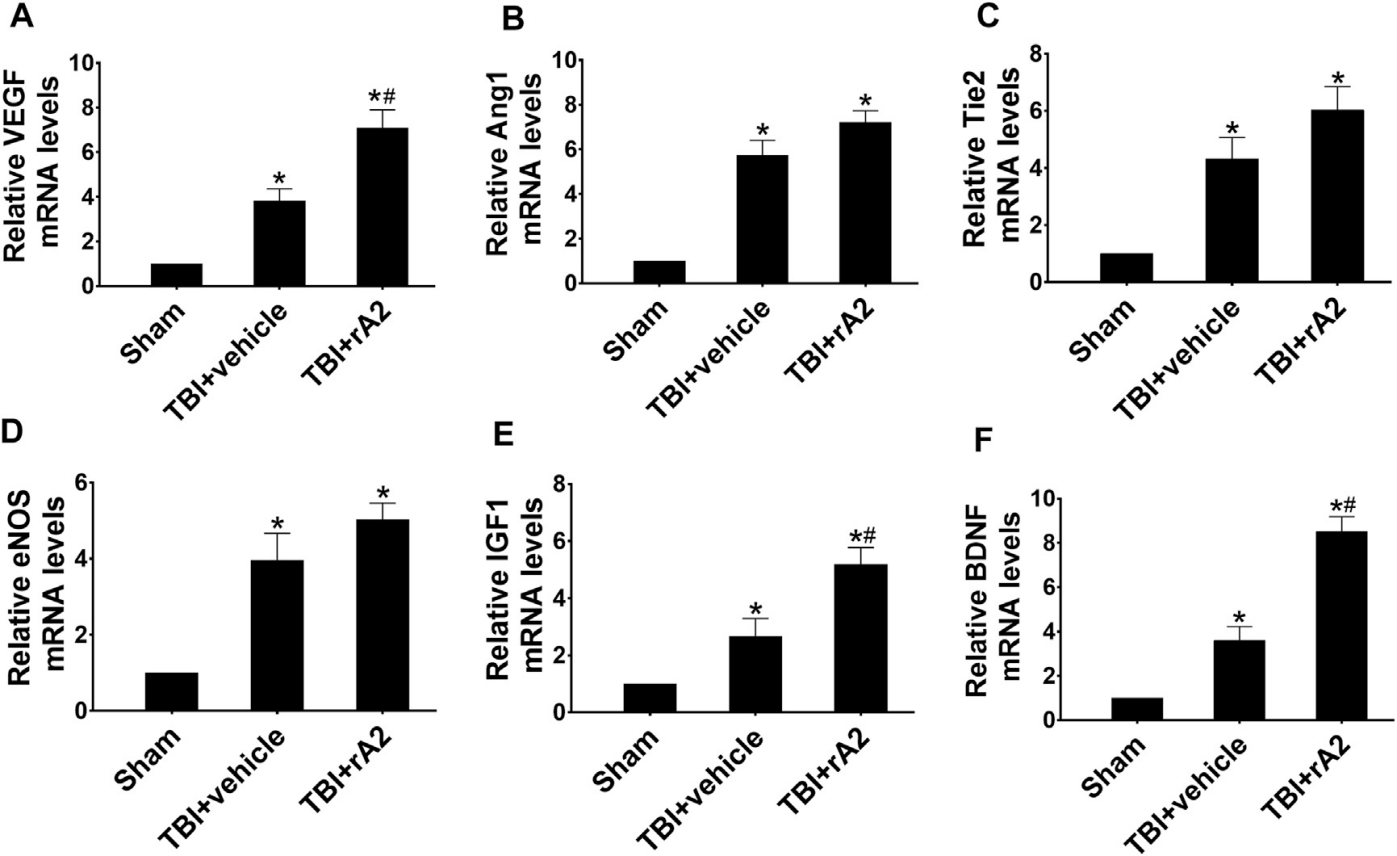
rA2 administration augments mRNA levels of pro-angiogenic factors in the isolated cerebral microvascular fragments at 7 days after TBI in mice. Gene expression levels of pro-angiogenic factors in the brain microvessels were measured at 7 days after TBI by RT-qPCR. Quantitative analysis of mRNA levels of VEGF **(A)**, Ang1 **(B)**, Tie2 **(C)**, eNOS **(D)**, IGF1 **(E)**, and BDNF **(F)**. Data are expressed as mean ± SEM; *n* = 4 mice per group, *p < 0.05 compared to Sham, ^#^ *p* < 0.05 compared to TBI+vehicle (1 mg/kg BSA).

To investigate the effects of rA2 administration in cerebrovascular remodeling at the delayed phase after TBI, we measured the expression of pro-angiogenetic and trophic factor genes of isolated cerebral microvascular fragments by using RT-qPCR at 7 days after TBI, including vascular endothelial growth factor (VEGF), angiopoietin1 (Ang1), Tie2, endothelial nitric oxide synthase (eNOS), insulin-like growth factor-1 (IGF-1), brain-derived neurotrophic factor (BDNF). The results showed, as expected, TBI significantly increased these gene expressions. However, rA2 treatment further augmented the gene expression of pro-angiogenetic and trophic factor genes VEGF, IGF, and BDNF in brain microvessels after TBI (Figure 2). Moreover, we further determined BDNF protein expression in the ipsilateral hemisphere at 3 and 7 days after TBI. Western blotting analysis showed that rA2 treatment significantly increased BDNF protein levels in the ipsilateral hemisphere at 3 and 7 days after TBI (Supplementary Figure 2). Taken together, these data indicating that rA2 may promote cerebral vascular remodeling and angiogenesis at the delayed phase after TBI.

### rA2 Treatment Significantly Induces CREB Signaling Pathway and Downstream Proteins, Including Vascular Endothelial Growth Factor and Brain-Derived Neurotrophic Factor in Human Brain Microvascular Endothelial Cells

**FIGURE 3:**
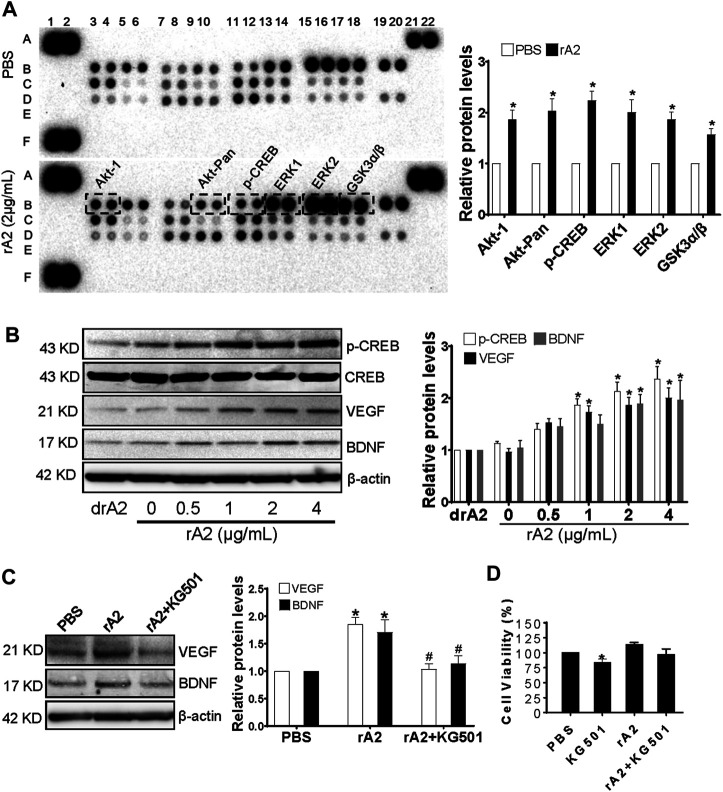
rA2 induces CREB phosphorylation, VEGF, and BDNF in HBEMC. **(A)** Representative MAPKs profile array and quantification in HBMEC treated with PBS or rA2 (2 μg/ml) for 24 h, *n* = 3 experiments. **(B)** Representative western blotting images of p-CREB, CREB, VEGF, BDNF, and β-actin and quantification for p-CREB, VEGF, BDNF in HBMEC treated with dose-dependent rA2 for 24 h *n* = 3 experiments. **(C)** Representative gel images and quantification of western blotting for VEGF, BDNF in HBMEC treated with rA2 (2 μg/ml) with or without KG-501 (10 μM) for 24 h. **(D)** Viability of cultured HBMEC after rA2 with/without KG501 treatment. *n* = 3 experiments. Data are expressed as mean ± SEM, *p < 0.05 compared to PBS, ^#^ *p* < 0.05 compared to rA2.

It has been reported that members of the MAPK family mediate CREB phosphorylation in endothelial cells (Mayo et al., 2001) and play a key role in regulating angiogenesis (Song and Finley, 2020). To investigate whether rA2-induced angiogenesis is associated with the activation of MAPKs and CREB, the MAPKs profile array was performed. The results showed that there is significant induction of p-Akt1 (S473), p-Akt-Pan (S472, S473, S474), p-CREB (S133), p-ERK1 (T202/Y204), p-ERK2 (T185/Y187), and p-GSK3α/β (S21/S9) expression in rA2 treated HBMEC (Figure 3A). CREB is the transcription factor that regulates vascular remodeling *via* mediating downstream growth factors, including VEGF (Kant et al., 2019). To analyze whether rA2 treatment induces the protein levels of p-CREB, VEGF, and BDNF in HBMEC, western blotting analysis was used. The results showed that treatment with 2 and 4 μg/ml of rA2 for 24 h significantly induces p-CREB, VEGF, and BDNF protein in HBMEC (Figure 3B). Next, we further determine whether CREB is responsible for rA2-induced VEGF and BDNF expression. HBMEC were treated with KG-501, a specific CREB inhibitor that blocks CREB transcription activation (Best et al., 2004). To expect, co-treatment with rA2 plus KG501 suppressed VEGF and BDNF protein levels (Figure 3C)**,** and has no significant effect on cell viability, compared with PBS or rA2 treatment group (Figure 3D). In all, these results suggested that rA2 treatment significantly induces CREB signaling pathway and downstream proteins, including VEGF and BDNF in HBMEC.

### rA2 Administration Enhances Cerebral Angiogenesis and Function Vessel Density After Traumatic Brain Injury in Mice

**FIGURE 4:**
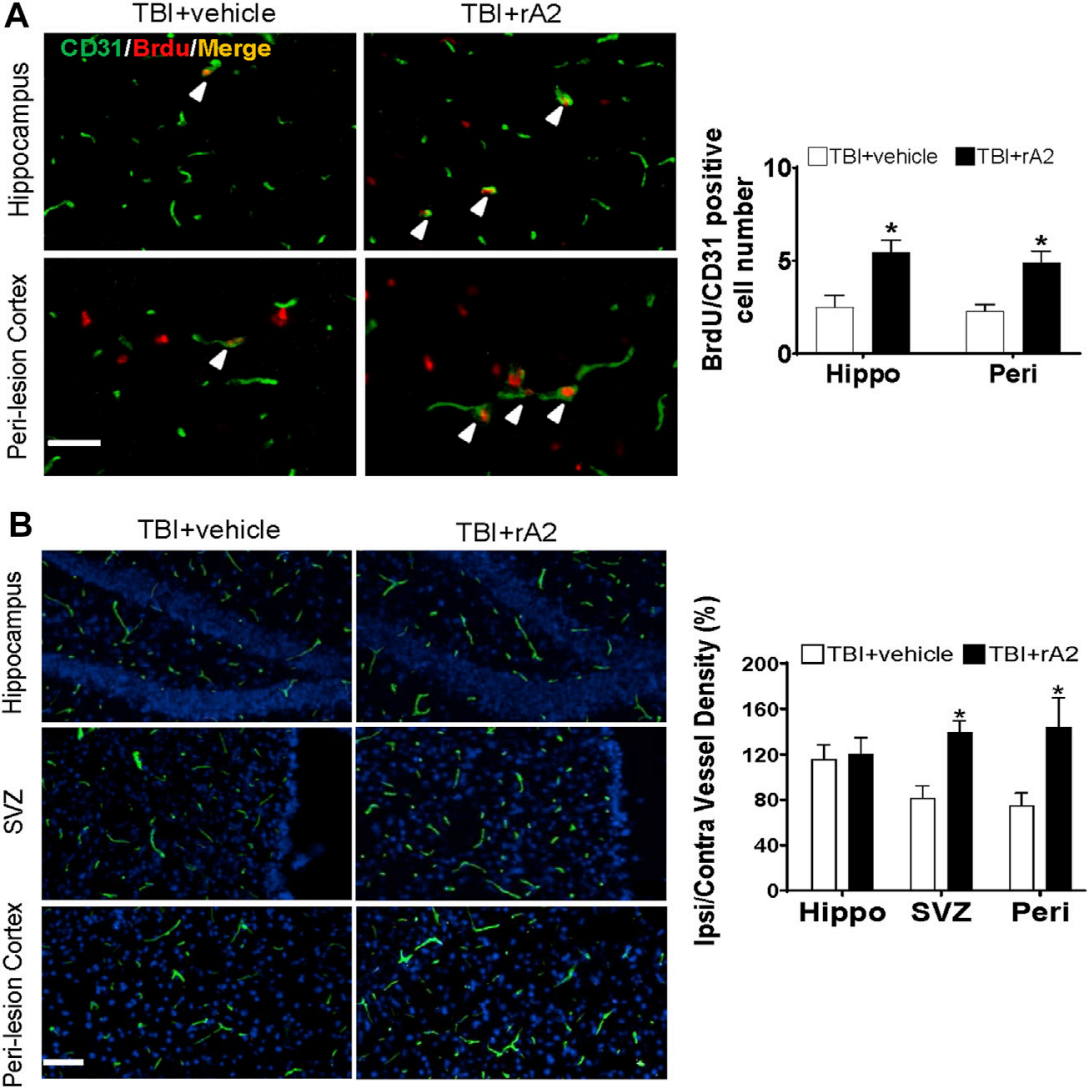
rA2 administration enhances cerebral angiogenesis and function vessel density after TBI in mice. Cerebral angiogenesis was determined at 14 days after TBI by bromodeoxyuridine (BrdU) and CD31 double immunostaining method, and functional vessel density was examined at 28 days by lectin perfused brain vessels analysis. **(A)**. Representative images and quantification of CD31^+^/BrdU^+^ cells (indicated by white arrows, cell number per filed) at hippocampus (Hippo) and peri-lesion cortex (Peri) areas of TBI mouse brains. **(B)**. Representative images of lectin and quantification of ipsilateral/contralateral vessel density (%) in the hippocampus (Hippo), SVZ, and peri-lesion cortex (Peri) areas of TBI mouse brains. Scale bar = 50 μm. Data are expressed as mean ± SEM, n = 6 mice per group. *p < 0.05 vs. TBI+vehicle (1 mg/kg BSA).

To further determine whether rA2 administration could induce cerebral vascular angiogenesis at the delayed phase after TBI, BrdU injection was initiated at 7 days after TBI and repeated daily for 7 days, the mice brain were collected at 14 days after TBI for co-immunostaining with BrdU and CD31. The results showed that the number of newborn vascular endothelial cells labeled with CD31+/BrdU+ was significantly increased in the hippocampus and peri-lesion area of rA2-treated TBI mice, compared with TBI mice (Figure 4A). Furthermore, after repeated daily rA2 treatment for 7 days, lectin dye (VECTOR, FL-1171) was intravenously injected at 28 days after TBI. Functional vessel density analysis showed that the ratio of ipsi/contra vessel density in the mice brain is significantly increased in the SVZ and peri-lesion areas (Figure 4B). Taken together, these results demonstrated that rA2 administration significantly improved the angiogenic process and increased functional vessel density in the peri-lesion areas of TBI mouse brains.

### rA2 Exposure Promotes Angiogenic Capability of Human Brain Microvascular Endothelial Cells *In Vitro*

To validate the role of rA2 in the promotion of angiogenic capability, after 24 h of hypoxia plus IL-1β insult (to mimic TBI injury at subacute/recovery phase) to the cultured HBMEC, we performed two independent *in vitro* assays of angiogenesis, matrigel tube formation, and scratch migration assays. The results of matrigel tube formation assay showed that hypoxia plus IL-1β for 24 h significantly reduces the number of tube formation by about 30% in the vehicle and deactivated rA2 (drA2) groups, compared to the vehicle-treated cells under normal condition, however rA2 exposure significantly increased the tube formation in HBMEC under normal and hypoxia exposure condition by about 55 and 70%, respectively (Supplementary Figure 3A). By using scratch assay, we found that hypoxia plus IL-1β insult has no significant inhibitory effect on the migration of HBMEC, but rA2 treatment induces significant gap closure of HBMEC under normal and hypoxia exposure condition by approximately 45% (Supplementary Figure 3B). Taken together, these results demonstrate that rA2 exposure promotes the angiogenic capability of HBMEC *in vitro*.

### rA2 Infusion Improves Neurological Outcomes and Reduces Brain Tissue Loss After Traumatic Brain Injury in Mice

**FIGURE 5:**
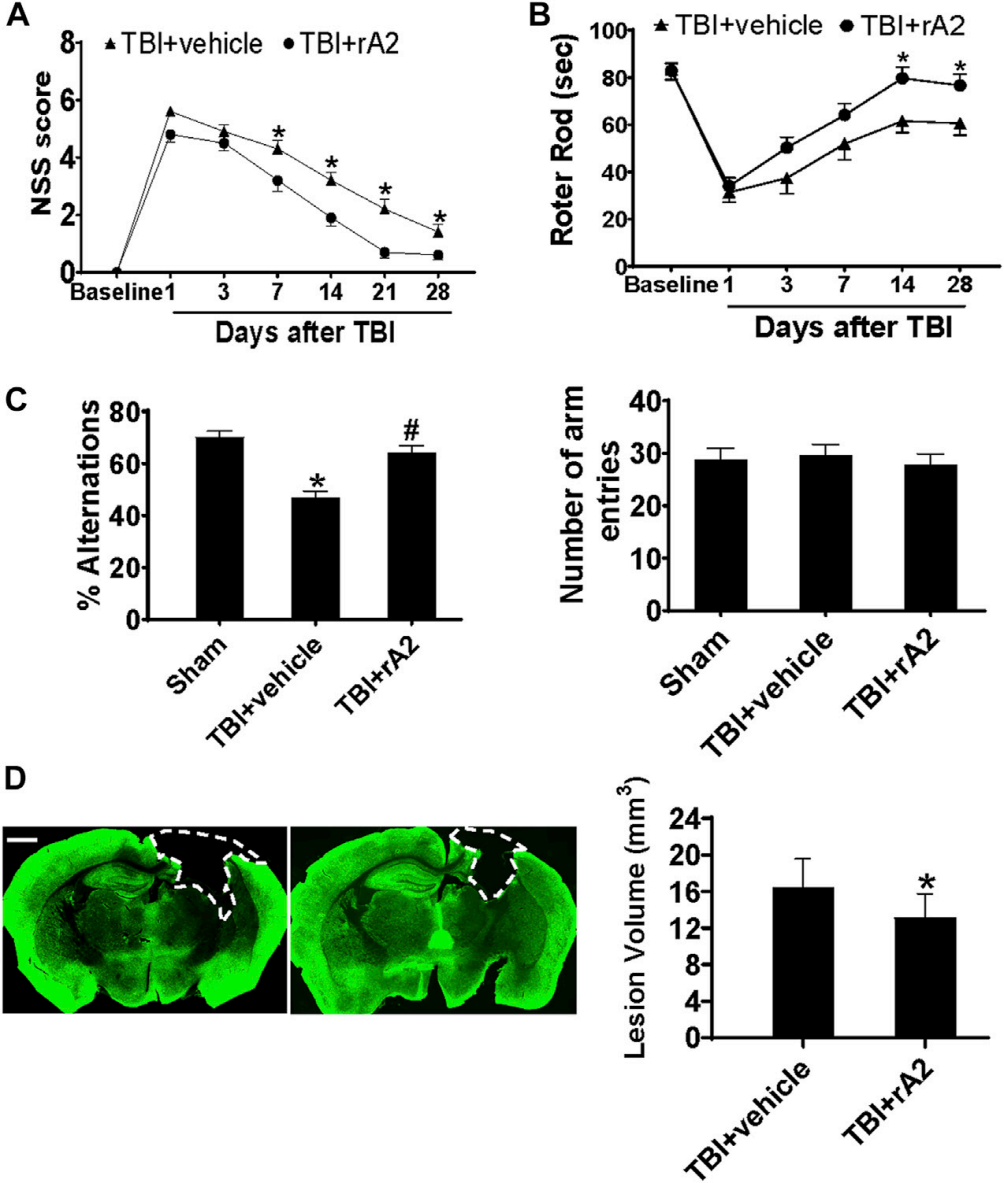
rA2 administration improves neurological outcomes and reduces brain tissue loss after TBI in mice. **(A)**. Modified neurological severity score (NSS). **(B)** Rotor rod test. *p < 0.05 compared to TBI+vehicle (1 mg/kg BSA) at the same time point. **(C)** Y-maze test was performed at 21 days after TBI. *n* = 14 mice per group. *p < 0.05 compared to Sham, ^#^ *p* < 0.05 compared to TBI+vehicle (1 mg/kg BSA). **(D)** Representative MAP2 staining and quantification of lesion volume. The dashed line indicates the border of the brain tissue loss. Scar bar, 1 mm *p < 0.05 compared to TBI+vehicle (1 mg/kg BSA). Data are expressed as mean ± SEM; *n* = 14 mice per group.

We next investigated whether rA2 daily treatment for 7 days could improve neurobehavioral outcomes after TBI. The sensorimotor function was evaluated *via* neurological severe score (NSS) and Rota-rod tests. The NSS test showed that rA2 treatment significantly improves neurological function from day 7 to day 28 after TBI (Figure 5A). The Rota-rod test also showed a significant improvement in the sensorimotor function by the rA2 administration at days 14 and 28 after TBI compared to the vehicle group (Figure 5B). We next investigated cognitive function at day 21 by the Y-maze test. The data showed that rA2 administration significantly increased the alternation ratio, but didn’t alter the total number of arm entries (Figure 5C). These neurobehavioral test results suggest that rA2 administration after TBI improves long-term motor and cognitive function recovery. Moreover, we further test the effect of rA2 treatment on brain tissue loss at 28 days after TBI by MAP2 staining. The results showed that rA2 daily treatment for 7 days significantly reduces brain tissue loss after TBI (Figure 5D). Taken together, these results suggested that rA2 administration improves neurological outcomes and reduces brain tissue loss after TBI in mice.

## Discussion

The major experimental findings are summarized in the followings: 1) rA2 administration initiated at 2 h after TBI significantly reduced early BBB disruption at 24 h after TBI in mice, which was associated with reduced down-regulation of BBB junction protein expression; 2) rA2 daily administration for 7 days significantly augmented mRNA levels of pro-angiogenic and endothelial-derived trophic factors in the isolated brain microvascular fragments at 7 days after TBI; 3) rA2 induces protein levels of VEGF and BNDF, which are mediated by CREB *in vitro*; 4) rA2 daily administration for 7 days also significantly increased cerebral angiogenesis at 14 days and vessel density at 28 days after TBI. 5) Additionally, in the cultured HBMEC, we validated that rA2 functions in the promotion of angiogenic capability. Lastly, we demonstrated that rA2 improves long-term sensorimotor ability and cognitive function, and reduces brain tissue loss at 28 days after TBI. Taken together, experimental results from the present study provided experimental evidence in support of our hypothesis, strongly suggest that rA2 may be developed as a novel and effective vascular targeting approach for treating TBI.

As early BBB breakdown results in increased vascular permeability, cerebral edema, inflammation, and neuronal cell death after TBI, the acute phase of BBB damage occupied a central place in TBI pathophysiology (Shlosberg et al., 2010). Therefore, resealing of the early BBB leakage is critical for attenuation of secondary brain injury, and has been proposed as a therapeutic intervention approach for treating TBI (Shlosberg et al., 2010; Chen et al., 2020). In the first set of experiments in TBI mice, we tested dose-range effects of rA2 administration given at 2 h after TBI in BBB integrity disruption examined with the commonly used Evans blue extravasation assay. Our results showed rA2 exhibits a significant protective effect in post-TBI BBB leakage in mice, and we identified that 1 mg/kg is the optimal dose of rA2. In the following experiment, we found rA2 showed a prolonged effective therapeutic time window in the prevention of BBB leakage up to 6 h after TBI. From our best knowledge, the present study, for the first time, demonstrated rA2 administration might have a potent BBB protection effect in animal models of TBI.

Although we did not propose mechanistic experiments in the present study, we might still speculate a few possible molecular mechanisms of rA2 BBB protection after TBI according to earlier experimental investigations from others and our group. For example, ANXA2 may function as endothelial membrane F-actin binding protein in bridging junction formation (Lee et al., 2004), and plays central roles in modulating tight junction integrity and BBB permeability (Hayes et al., 2009; Grieve et al., 2012). A previous study found ANXA2 functions to maintain pulmonary microvascular integrity, and prevents vascular leak during alveolar hypoxia by enabling vascular endothelial cadherin-related phosphatase activity (Luo et al., 2017). Our group recently demonstrated that ANXA2 is an essential membrane protein that contributes to BBB development, particularly in BBB formation and function by regulation of cerebral endothelial cell-to-cell junctions. In a cell culture injury model, we also demonstrated rA2 could ameliorate hypoxia plus IL-1β insult-induced transendothelial permeability increase (Li et al., 2019), and these protective effects of rA2 are mediated in part *via* F-actin/VE-cadherin interactions to strengthen endothelial tightness, and modulating Robo4-paxillin-ARF6 signaling pathway (Li et al., 2019).

Importantly, the central substrate for the BBB *in vivo* is still centered on the regulation of adherens and tight junctions between cerebral endothelial cells (Ronaldson and Davis, 2012). Therefore, in this study, we investigated the effect of rA2 administration in the expression of endothelium tight junction protein ZO-1, occludin, and claudin-5, and adheren protein VE-Cadherin, which are required for the assembly of BBB architecture (Tietz and Engelhardt, 2015). We found that rA2 administration significantly upregulated the protein levels of ZO-1 and VE-Cadherin, but not occludin, and claudin-5, in the ipsilateral brain tissue of TBI mice. These data suggest that up-regulation of BBB junction protein expression might at least in part contribute to the BBB protection of rA2 after TBI. However, the detailed underlying molecular mechanisms of the rA2 BBB protection need to be elucidated in the animal TBI models in the future.

The cerebrovascular system plays a crucial role in maintaining CNS homeostasis (Segarra et al., 2021). Starting from the subacute phase to the recovery phase after TBI, cerebrovascular remodeling is one of the most important endogenous repair processes for recovery (Salehi et al., 2017). Cerebrovascular endothelial-derived trophic factor as niche support to prevent neurodegeneration, and cerebral angiogenesis to promote neurorepair are the two of most important mechanisms to boost recovery after TBI (Lee et al., 2009; Salehi et al., 2017). In the second set of experiments with the TBI mouse model, we examined the effects of rA2 daily administration for 7 days in the expression of cerebrovascular endothelial-derived trophic/pro-angiogenic factor gene expressions using RT-qPCR. The results showed rA2 significantly increases mRNA levels of VEGF-A, Ang1, Tie2, eNOS, IGF-1, and BDNF in the isolated cerebral microvessels of the ipsilateral brain at 7 days after TBI, and rA2 treatment significantly increases BDNF protein levels in the ipsilateral hemisphere at 3 and 7 days after TBI. These data suggested the rA2-administration may elevate trophic/pro-angiogenic factor expression and releasing as niche support to prevent neurodegeneration, as well as promote angiogenesis and neurorepair. Previous experimental investigations support our earlier speculation. For example, VEGF-A and its receptors initiate the formation of immature vessels, while Ang1 and other molecules play essential roles in regulating maturation, stabilization, and remodeling of vessels (Yancopoulos et al., 1998). Pro-angiogenetic factors VEGF-A/VEGFR-2 plays a key role in enhancing angiogenesis during the recovery phase after TBI *via* activating multiple pro-angiogenetic signaling pathways (Carmen-Orozco et al., 2019). Angiopoietin/Tie2 axis mediates neuroprotection and BBB stability in juvenile mice after TBI (Brickler et al., 2018). In addition, eNOS is a downstream mediator of VEGFR-2 and is critical for angiogenesis after TBI (Wu et al., 2011). IGF-1 and BDNF are well-known angiogenic factors, but both IGF-1/IGF-R and BDNF signaling also play key roles in promoting neuronal repair and neurological outcomes after TBI (Madathil and Saatman, 2015). Importantly, through MAPKs array, we found that rA2 treatment considerably augments p-Akt1 (S473), p-Akt-Pan (S472, S473, S474), p-CREB (S133), p-ERK1 (T202/Y204), p-ERK2 (T185/Y187), and p-GSK3α/β (S21/S9) expression, and further validate that CREB signaling mediates rA2-induced VEGF and BDNF expression in HBMEC. Consistent with our data, a previous study has reported that members of the MAPK family such as ERK, Akt, PKC mediated CREB phosphorylation in endothelial cells (Mayo et al., 2001). Interestingly, CREB is the key transcription factor that positively regulates VEGF (Kant et al., 2019), and BDNF (Tao et al., 1998) in the brain, which is responsible for the recovery of spatial learning and memory function after TBI (Griesbach et al., 2004). Therefore, in combination with others and our data, rA2 may induce angiogenesis and vascular remodeling, at least in part, through CREB-mediated VEGF and BDNF upregulation in brain endothelial cells after TBI. However, the role of rA2 in regulating cerebrovascular endothelium-derived trophic/pro-angiogenic factors after TBI warrants further investigation in the future.

Emerging investigations have demonstrated that angiogenesis is fundamental to normal brain development, and repair as well after brain injury. It has been suggested that cerebral angiogenesis is one of the most essential and critical mechanisms for functional recovery after TBI (Salehi et al., 2017). Indeed, a body of evidence has already early indicated that ANXA2 may function in promoting angiogenesis in the tumor, retinal, corneal, and brain tissues (Zhai et al., 2011; Liu and Hajjar, 2016). By co-immunostaining with BrdU and CD31, our experimental results showed rA2 administration significantly increased the number of newborn vascular endothelial cells labeled with BrdU+/CD31+ at 14 days after TBI, and functional vessel density at 28 days after TBI at the hippocampal and peri-lesion area of mouse brains. Furthermore, we also validated the effects of rA2 in promoting the angiogenic capability of HBMEC *in vitro*.

In the third set of experiments, we performed neurobehavioral assessments, and the experimental results showed that rA2 administration significantly improves sensorimotor and cognitive function up to 28 days after TBI. Taken together, all experimental results demonstrated that rA2 administration significantly inhibited early BBB integrity disruption, and in the subacute phase, rA2 administration also significantly improved cerebrovascular remodeling, particularly in endothelium-derived pro-angiogenic/trophic factor expression, and cerebral angiogenesis and microvessel density after TBI in mice. Thereby the cerebrovascular effects of rA2 might prevent neurodegeneration and promote neurorepair, and ultimately improve long-term neurological outcomes after TBI.

We are aware that the present study has several limitations. Firstly, this is a proof-concept pilot study, we demonstrated rA2 administration could improve the neurological outcome of TBI in mice, which was associated with early BBB integrity disruption reduction, and cerebrovascular remodeling promotion at subacute and recovery phases. However, the causal relation of the pathological cerebrovascular effects by rA2 administration with the improved long-term neurological outcomes needs to be defined and validated in different TBI animal models. Secondly, the mechanistic insights of cerebrovascular effects by rA2 administration warrens more investigations in the future. Thirdly, from the therapeutic approach development perspective, all translational aspects of rA2 administration for treating TBI require careful preclinical evaluation.

In summary, in the present study, our experimental results showed that rA2 administration (1 mg/kg) significantly reduced early BBB disruption at 24 h after TBI. Moreover, we found that rA2 daily treatment for 7 days augmented TBI-induced mRNA levels of pro-angiogenic and endothelial-derived trophic factors in brain microvascular fragments. Consistently, rA2 treatment significantly increased cerebral angiogenesis examined at 14 days and vessel density at 28 days after TBI. Lastly, we demonstrated that rA2 administration improves long-term sensorimotor and cognitive function, and reduces brain tissue loss at 28 days after TBI. Our experimental findings strongly suggest that rA2 administration might be developed as a novel and effective cerebrovascular targeting strategy for treating TBI. Further investigations in the understanding of these mechanisms underlying annexin A2-cerebral vascular integrity and remodeling signaling pathways may lead to new strategies to treat CNS injury and diseases.

## Data Availability

The original contributions presented in the study are included in the article/Supplementary Material, further inquiries can be directed to the corresponding authors.

## Supplementary Material

Supplementary Figure 1 Plasma concentration of Annexin A2. The plasma concentrations of Annexin A2 were quantified at 0, 2, 6, 12, 24, 48, and 72 h post-injection (i.p.) by performing ELISA by using specific calibration curves. The concentrations are expressed as micrograms of Annexin A2 per milligram of the plasma and are represented as means and SEM. *n* = 3 mice per time point.

Supplementary Figure 2 rA2 administration increases protein levels of BDNF in the ipsilateral hemisphere after TBI. Representative gel images and quantification of western blotting for BDNF in ipsilatral brain of mice treated with rA2 (1 mg/kg) or vehicle (BSA, 1 mg/kg) at 3 and 7 days after TBI. *n* = 5 mice per group. Data are expressed as mean ± SEM, **p* < 0.05 compared to Sham, ^#^ *p* < 0.05 compared to BSA.

Supplementary Figure 3 rA2 exposure promotes angiogenic capability of HBMEC *in vitro.* The effects of rA2 exposure at 2 µg/ml for 24 h in the number of tube formation, and enhanced endothelial migration in cultured HBMEC. **(A)** Representative images of tube formation and quantification of tube numbers in the cultured HBMEC. Data are expressed as mean ± SEM, *n* = 6 wells per group, **p* < 0.05 compared with PBS under normal condition, ^#^ *p* < 0.05 compared with rA2 under normal condition. **(B)** Representative images of scratch migration assay and quantification of gape closure (%) in HBMEC. Data are expressed as mean ± SEM, n = 5 wells per group, **p* < 0.05 compared with PBS treated controls.
