# Supplementary figures and images for: Recombinant Annexin A2 Administration Improves Neurological Outcomes After Traumatic Brain Injury in Mice
Source: Front Pharmacol. 2021 Jul 12;12:708469. doi: 10.3389/fphar.2021.708469 (PMC8363504; doi:10.3389/fphar.2021.708469)

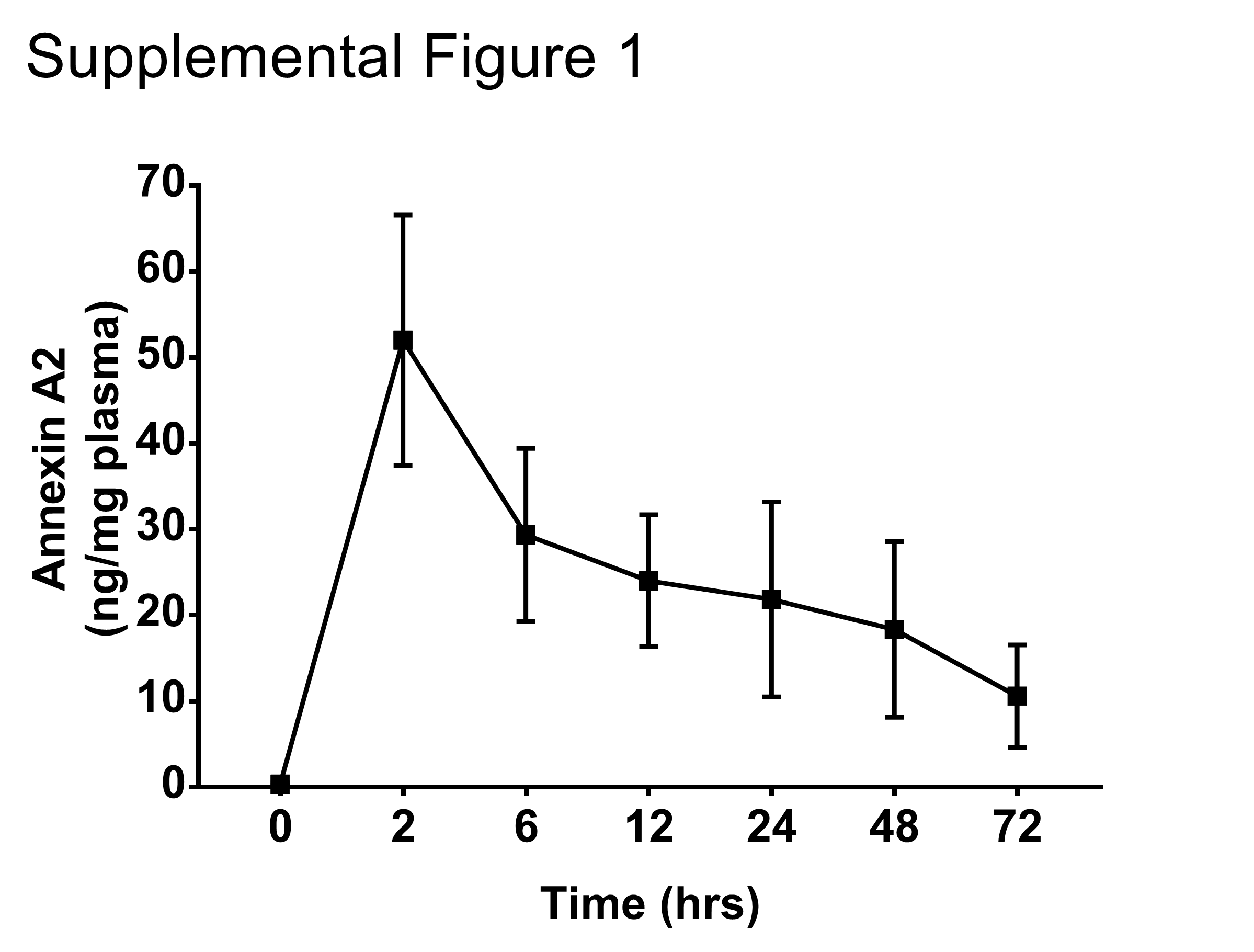

Supplement: Supplementary Figure 1 — Plasma concentration of Annexin A2. The plasma concentrations of Annexin A2 were quantified at 0, 2, 6, 12, 24, 48, and 72 h post-injection (i.p.) by performing ELISA by using specific calibration curves. The concentrations are expressed as micrograms of Annexin A2 per milligram of the plasma and are represented as means and SEM. n = 3 mice per time point. [file Image1.tif]

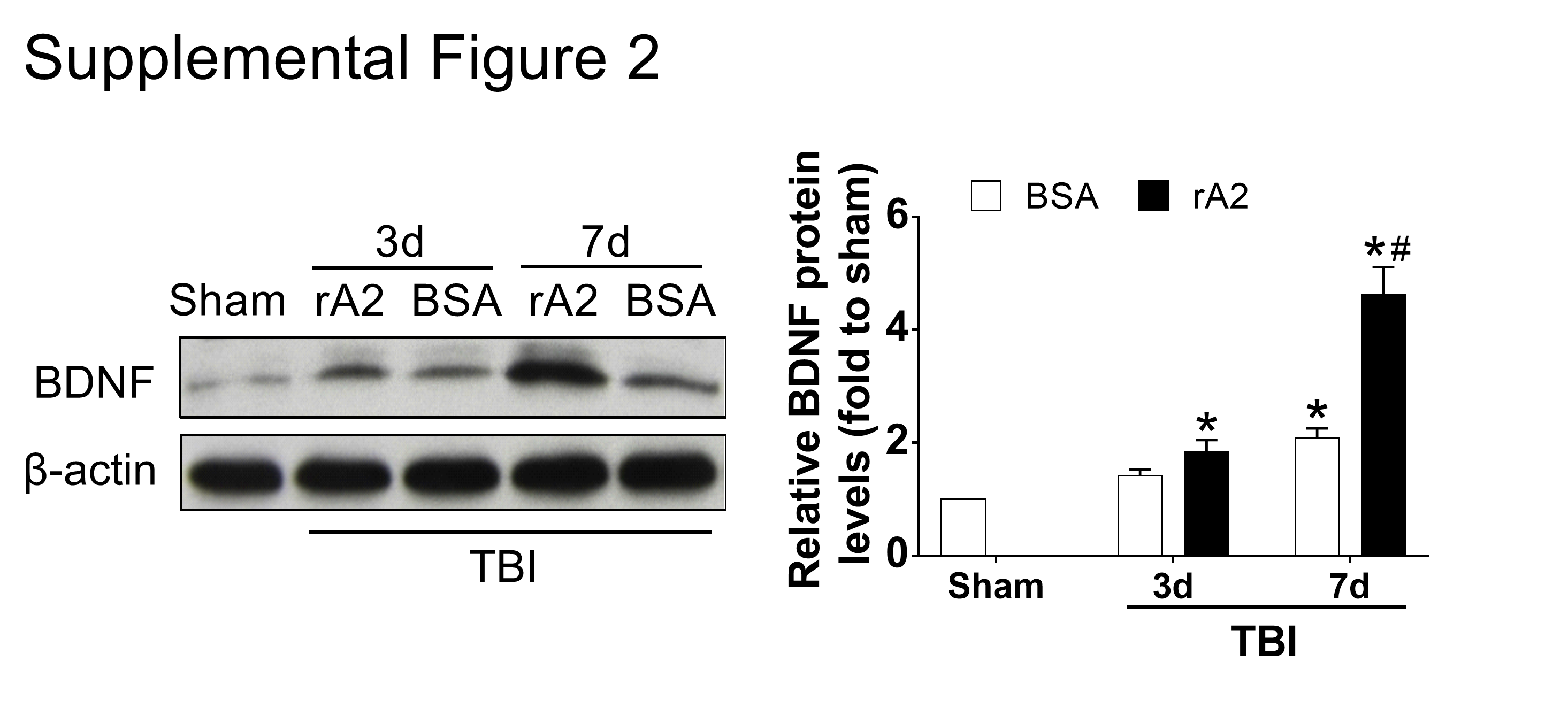

Supplement: Supplementary Figure 2 — rA2 administration increases protein levels of BDNF in the ipsilateral hemisphere after TBI. Representative gel images and quantification of western blotting for BDNF in ipsilatral brain of mice treated with rA2 (1 mg/kg) or vehicle (BSA, 1 mg/kg) at 3 and 7 days after TBI. n = 5 mice per group. Data are expressed as mean ± SEM, *p < 0.05 compared to Sham, # p < 0.05 compared to BSA. [file Image2.tif]

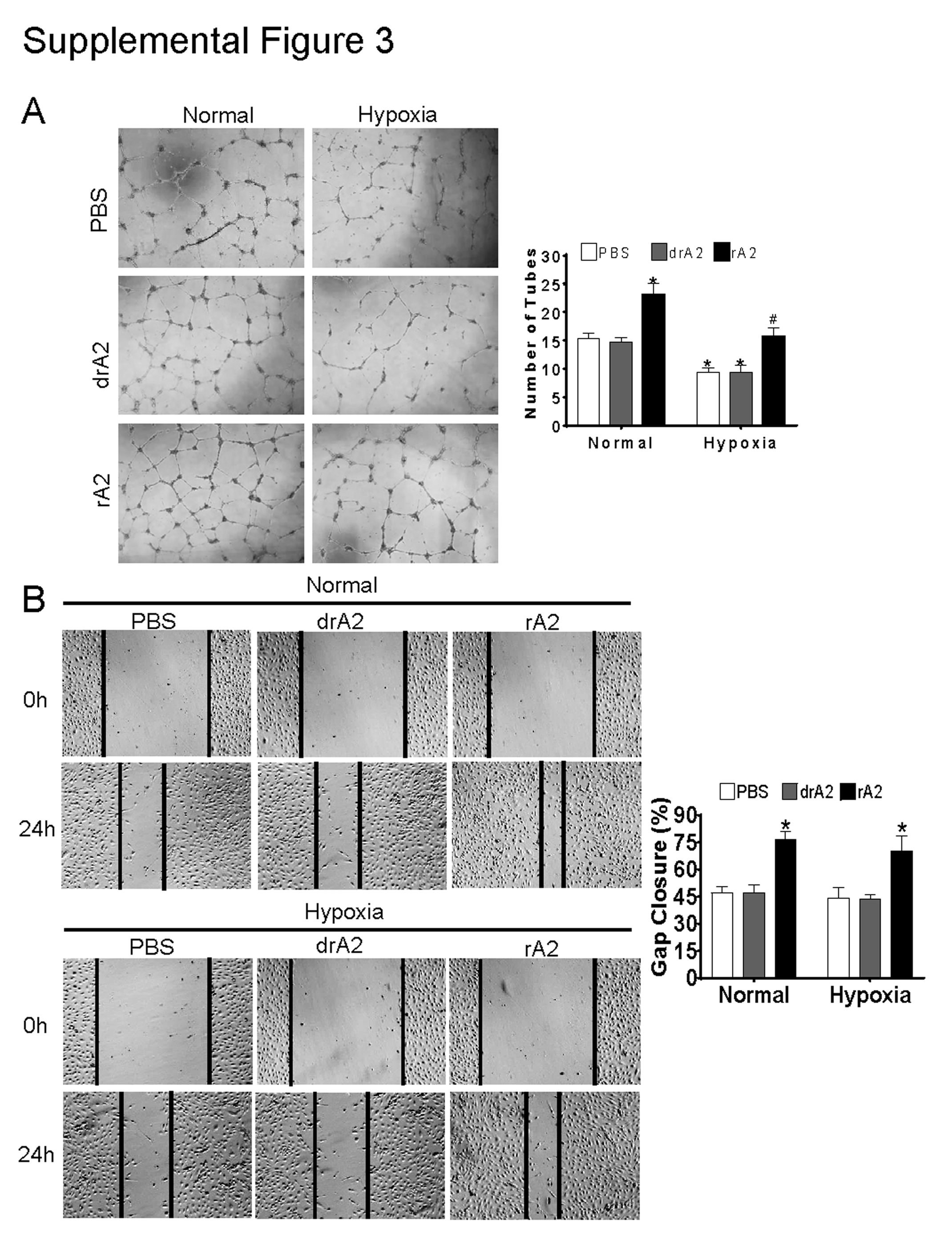

Supplement: Supplementary Figure 3 — rA2 exposure promotes angiogenic capability of HBMEC in vitro. The effects of rA2 exposure at 2 µg/ml for 24 h in the number of tube formation, and enhanced endothelial migration in cultured HBMEC. (A) Representative images of tube formation and quantification of tube numbers in the cultured HBMEC. Data are expressed as mean ± SEM, n = 6 wells per group, *p < 0.05 compared with PBS under normal condition, # p < 0.05 compared with rA2 under normal condition. (B) Representative images of scratch migration assay and quantification of gape closure (%) in HBMEC. Data are expressed as mean ± SEM, n = 5 wells per group, *p < 0.05 compared with PBS treated controls. [file Image3.tif]
